# Thinking Against Burnout? An Individual’s Tendency to Engage in and Enjoy Thinking as a Potential Resilience Factor of Burnout Symptoms and Burnout-Related Impairment in Executive Functioning

**DOI:** 10.3389/fpsyg.2019.00420

**Published:** 2019-03-12

**Authors:** Monika Fleischhauer, Robert Miller, Magdalena Katharina Wekenborg, Marlene Penz, Clemens Kirschbaum, Sören Enge

**Affiliations:** ^1^Department of Psychology, Brandenburg Medical School, Neuruppin, Germany; ^2^Department of Psychology, Technische Universität Dresden, Dresden, Germany; ^3^Department of Psychology, Faculty of Natural Sciences, Medical School Berlin, Berlin, Germany

**Keywords:** burnout, personality, need for cognition, Big Five, executive functions

## Abstract

The personality trait need for cognition (NFC) refers to individual differences in the tendency to engage in and enjoy cognitive endeavors. In today’s working world, which is characterized by increasing cognitive demands, NFC may contribute to resilience against work-related stress and burnout symptoms. We investigated this question in a large population-wide sample of 4,134 individuals (Study 1) and in a sample of 125 students (Study 2). NFC was consistently negatively related to the burnout facets emotional exhaustion and reduced personal efficacy of the Maslach burnout inventory and explained up to 10% additional variance in burnout symptoms over and above the five-factor model of personality. In the student sample, where stress factors are mainly cognitive in nature, NFC was the most relevant predictor. In this sample, we additionally investigated whether NFC might be a relevant moderator of the inconsistently found associations between burnout and impairments in cognitive functioning. The participants conducted three cognitive tasks (number–letter task, two-back task, and Go/NoGo task) that measure the executive functions switching, updating, and response inhibition, respectively. While burnout was slightly negatively related to working memory performance, NFC did not moderate the relationship between burnout and executive control which could be traced back to the young and healthy sample used to examine this research question. All in all, our results clearly suggest that NFC may be an important individual difference factor contributing to the resilience against burnout, especially if stress factors are cognitive in nature.

## Introduction

Work-related chronic stress is a major health challenge in Western societies. The World Health Organization assumes work stress as a growing health risk for both, the individual and the society (Leka and Jain, 2010). Within the German Health Interview and Examination Survey for Adults (DEGS1) conducted between 2008 and 2011, 11% of the 5.850 participants between 18 and 64 years reported high levels of chronic stress. Higher levels occurred for woman than for men and for those with lower socio-economic status (Hapke et al., 2013).

One phenomenon often described in connection with chronic stress at work is burnout. Burnout was originally attributed to the field of social work, arguing that employees of the social work sector may develop burnout due to the highly emotional demands of client-work (Maslach and Jackson, 1981). About a quarter century later, the definition of burnout-causing working environments is broaden, so that any kind of work is now potentially considered to bear a risk of burning out (Schaufeli and Taris, 2005), with an estimated prevalence of 13% (Norlund et al., 2010) to 26% (Adriaenssens et al., 2015) in the general working population of Western countries and with an increasing trend at least for some professions (Shanafelt et al., 2015).

By definition, the burnout syndrome encompasses three dimensions, namely emotional exhaustion (EE), cynical attitudes toward work (CY), and reduced personal efficacy (rPE) (Maslach et al., 2001). EE is characterized as depletion of emotional resources and feelings of being overextended by work. CY refers to a mental distancing from work. Finally, rPE is defined as negative evaluation of one’s own work, leading to feelings of insufficiency and poor work-related self-esteem (for an overview, see Schaufeli and Salanova, 2014). Following Schaufeli and Taris (2005), EE and CY constitute the core dimensions of burnout, consequently leading to rPE.

Burnout is considered a major risk factor for impaired health status such as the cardiovascular diseases (Toker et al., 2012; Leiter et al., 2013). However, cross-sectional designs show a large syndrome overlap with depression (Bianchi et al., 2015). Therefore, at the time, the classification systems DSM-V (Diagnostic and Statistical Manual of Mental Disorders; American Psychiatric Association, 2013) and ICD-10 (International Classification of Disease; World Health Organization, 1992) do not include burnout as an independent clinical diagnosis. Nonetheless, in the ICD-10, burnout is mentioned within the residual category Z 73 – problems related to life management difficulty. Currently, two examples for standardized burnout diagnosis exist: The Swedish and the Dutch health systems provide standardized diagnoses that integrate burnout as a definable syndrome (Van Der Klink and Van Dijk, 2003; Schaufeli et al., 2009).

The enormous individual suffering of all those affected as well as the escalating economic costs associated with an increase in burnout prevalence (e.g., Shanafelt et al., 2015) justify intensified research effort invested in the identification of etiological and protective factors potentially associated with burnout. An empirically validated model of burnout determining factors is a necessary prerequisite for the development of effective prevention and treatment strategies. Etiological factors of burnout can broadly be categorized into individual factors (socio-demographic and personality characteristics) and situational factors (job, organizational, and occupational characteristics) (Maslach et al., 2001; Kaschka et al., 2011). Given the fact that burnout is per definition an individual experience that is specific to the work context, it does not seem surprising that since the introduction of the burnout concept in the 1970s research mostly focused on occupational conditions. During the last decade, however, research increasingly addressed individual difference factors that may moderate the vulnerability and resilience to burnout. In this regard individual differences in personality have been considered particularly important (Armon et al., 2012), with most of the studies referring to the Five-factor model (FFM) of personality.

In terms of the FFM, two large-scale meta-analyses revealed associations of the FFM with all three burnout dimensions (Alarcon et al., 2009; Swider and Zimmerman, 2010). Neuroticism (N), characterized by hypersensitivity, moodiness, and depression, has been shown to exhibit consistently strong positive relationships with EE and moderate associations with CY and rPE. Higher scores in Extraversion (E), defined as being sociable, energetic, outgoing, and optimistic, showed significant moderate negative associations with all burnout dimension. Small to moderate negative correlations with the burnout dimensions were also observed for Agreeableness (A), characterized by altruism, empathy, and cooperation, as well as for Conscientiousness (C), broadly characterized by competence, dutifulness, and self-discipline. For Openness (O) that is linked to curiosity and the willingness to try new things and ideas, both meta-analyses revealed moderate negative associations with the burnout dimension rPE (Alarcon et al., 2009; Swider and Zimmerman, 2010), but only weak (Swider and Zimmerman, 2010) or non-significant (Alarcon et al., 2009) negative associations with CY and EE. This pattern has also been interculturally validated by a meta-analysis focusing on Chinese samples (You et al., 2015).

Additionally, although to a lesser extent, other personality factors have been studied in their relation to burnout: Positive associations were found with (maladaptive) narcissism (e.g., von Känel et al., 2017) and negative affectivity, whereas self-esteem, self-efficacy, internal locus of control, positive affectivity, optimism, proactive personality, and hardiness depicted small to moderate negative associations with burnout (Alarcon et al., 2009).

However, there is little work on personality traits that refer to intrinsic cognitive engagement or on so-called cognitive investment traits. Why might this be important? At the globalized market, companies have to adapt quickly to changing environments. Work is getting more and more complex and rather cognitively than physically demanding, for example, due to the growth of technology and digitalization, which also requires increasing flexibility and cognitive engagement from employees. Increasing cognitive demands in the working environment may also be seen as one explaining factor for the heightened relevance of burnout at the job market. And indeed, individuals with burnout often report to have difficulties with attention, concentration, and memory. In this context, Deligkaris et al. (2014) reviewed studies in the realm of cognitive factors and burnout, and found a negative association between burnout and objectively measured cognitive functioning (executive functions, attention, and memory) in 13 of the 15 reviewed studies. For example, comparing no-burnout controls with clinical burnout cases, van der Linden et al. (2005) could show that high levels of burnout symptoms are associated with difficulties in response inhibition and sustained attention. Moreover, Jonsdottir et al. (2013) found impaired memory performance in clinical burnout individuals compared to healthy controls. Considering non-clinical samples, results appear less clear and dependent on moderating factors. Using a population-based sample of young adults, Castaneda et al. (2011) found no evidence for a negative association between burnout symptoms and executive control, whereas Diestel et al. (2013) reported evidence for a negative association between burnout and executive functions, however, only when task demands are high. Also using a non-clinical sample, a more recent study of Bianchi et al. (2018) contributed to the research field by reporting that individuals with higher burnout scores did not differ from those with lower burnout scores in overall memory performance but showed increased recall of negative words and decreased recall of positive words. All in all, the growing body of research suggests that burnout is associated with chronic impairments of executive control, but that in non-clinical samples where burnout symptoms are still less severe, the relationship between burnout and cognitive impairments may be moderated by situational characteristics. In this context, it is still unclear whether and to what extent stable individual differences in personality may modulate this relationship. One personality factor that is related to individual differences in cognitive engagement/investment and that could contribute to resilience against the development of burnout and against burnout-related impairments in cognitive control is the need for cognition (NFC).

Need for cognition that was originally conceptualized by Cacioppo and Petty (1982) refers to individual differences in intrinsic cognitive motivation, that is “an individual’s tendency to engage in and enjoy effortful cognitive endeavors” (Cacioppo et al., 1996, p. 197). Low and high levels in NFC were conceived as opposite ends of a continuum with low NFC being defined as the relative absence of an individual’s stable intrinsic motivation to engage in cognitive endeavors and effortful cognitive processing. Accordingly, individuals high in NFC were characterized as “chronic cognizers” who “naturally tend to seek, acquire, think about, and reflect back on information to make sense of stimuli, relationships, and events in their world” (Cacioppo et al., 1996, p. 198). In contrast, individuals low in NFC were considered as “cognitive misers” who are more likely to rely on cognitive shortcuts (e.g., heuristics) or others’ opinions (e.g., experts) to make sense of their world and typically invest less cognitive resources in cognitive and decision-making task. This characterization includes that individual differences in NFC are not limited to specific topics, but are rather defined at a macro-level referring to the enjoyment of cognitive activity or cognitive demands in general (Cacioppo et al., 1996). The definitions’ phrase “*to engage in and enjoy*” further illustrates that the motivation to think is rather process- than outcome-oriented, that is, individuals high in NFC draw their gratification from the act rather than from the result of thinking. Moreover, as NFC relates to the motivation to invest cognitive effort, it is thought to be related to, but also relatively distinct from cognitive abilities (i.e., intelligence). Support for the latter was found by several studies showing that NFC is slightly to moderately associated with aspects of intellectual ability and academic achievement (e.g., Fleischhauer et al., 2010; Hill et al., 2013). That cognitive motivation rather than intellectual ability is the key difference between low and high NFC individuals is also supported by research showing that extrinsic reward (Thompson et al., 1993) or strong personal relevance (Axsom et al., 1987) may stimulate low NFC individuals to engage similarly as individuals high in NFC in cognitive challenges.

Proceeding from these findings, individuals high in NFC should also enjoy cognitive tasks in their daily working environment and may also better resist stress related to cognitively demanding tasks. In this context, studies show that individuals high in NFC prefer complex to simple tasks (Cacioppo and Petty, 1982) and that the mere labeling of a message as being complex already motivated individuals high in NFC to engage in elaborated information processing (See et al., 2009; Fleischhauer et al., 2015). Small to moderate positive associations were also observed between NFC and personality traits indicating goal-orientation such as Persistence and C (Fleischhauer et al., 2010), illustrating that they may be better able to cope with cognitively challenging situation, also in the long run. This in turn may also result in their higher self-reported gratification from thinking (e.g., Cacioppo and Petty, 1982; See et al., 2009), higher self-esteem (e.g., Elias and Loomis, 2002), and emotional stability (e.g., Sadowski and Cogburn, 1997; Fleischhauer et al., 2010). All in all, the reported core conceptual aspects and behavioral correlates of NFC suggest that NFC could be an important individual difference factor potentially providing resilience to develop burnout symptoms.

Moreover, there is evidence that NFC might be a relevant moderator of the relationship between burnout symptoms and cognitive impairments. With respect to depression that shows large syndrome overlap with burnout (Bianchi et al., 2015), for example, Nishiguchi et al. (2016) reported a direct effect of depressive symptoms on the self-reported efficiency of executive attention (EC) and an indirect effect of NFC on this relationship. That is, individuals with higher levels of NFC were less likely to show cognitive impairments caused by depressive symptoms. These results were also supported by longitudinal path analyses of the authors demonstrating that cognitive motivation at T1 predicts EC on T2 and that a higher EC buffers depressive symptoms. Although in this study self-reports of cognitive functions were used and thus, the observed associations might be partly due to shared method-variance, it demonstrates the potential of NFC as protecting factor from experiencing negative behavioral effects of stress.

## The Present Research

In the present research, we examined whether NFC is negatively associated with burnout symptoms as measured with the widely used Maslach burnout inventory (MBI) and whether the correlations may change when controlling for variance of the personality factors of the FFM that were found to be related to burnout dimensions (Alarcon et al., 2009; Swider and Zimmerman, 2010) and that also share some variance with NFC (e.g., Fleischhauer et al., 2010). In *Study 1*, these bi- and multivariate associations were assessed in a large population-wide sample of 4,134 participants. In *Study 2*, we examined whether the associations between NFC and burnout facets were similarly or were even more pronounced in a sample of students that are relatively homogenous regarding age, occupation, and life circumstances.

Moreover, the student sample served to examine whether intrinsic cognitive motivation as depicted by NFC could serve as a moderator of the relationship between burnout and cognitive performance (Deligkaris et al., 2014). Here we followed the theoretical model of Miyake et al. (2000) that traces performance differences in cognitive tasks back to the three interrelated but inherently independent constructs “task shifting,” “updating/working memory,” and “response inhibition.” For these executive control functions also negative associations with burnout have been reported (for an overview, see Deligkaris et al., 2014). Therefore, cognitive performance in three tasks each measuring one of these executive function were assessed and the relationship to burnout symptoms as well as the moderating role of NFC for the relationship between burnout and executive control were analyzed. We hypothesized that impaired cognitive performance going along with burnout symptoms would especially occur for individuals low in NFC, whereas those high in NFC may be more able to compensate for burnout-related impairments.

## Study 1

### Materials and Methods

#### Participants and Procedure

Data were collected via online-assessment within a population-based German-speaking sample of 4.134 participants. After the participants had given written informed consent, socio-demographic variables were assessed such as age, sex, family and financial status, persons per household, education, and work status. Afterward participants completed a series of questionnaires for about 35 min. For the present study, only the questionnaires measuring burnout, NFC, and the FFM are relevant and described in the following. After finishing the test battery, participants got feedback about their personal burnout risk. Additionally, they could read information about burnout prevention or help in finding professional assistance.

The sample had a mean age of 42.5 years (*SD* = 10.9 years, range 18–70 years) and women were overrepresented (64.9%). About 90% of the sample were currently employed and the mean working hours per week were 40.6 (*SD* = 10.6, range = 0–110) h. When individuals were asked about the type of their work by a three-categorical variable, 80% reported to be “mainly cognitively engaged” at work, whereas the remaining 20% reported to be “mainly physically engaged” (2%) or “both physically and cognitively engaged” (18%).

#### Measures

The German version of the MBI-General Survey (MBI-GS, Büssing and Glaser, 1999) was used to assess the burnout dimensions *EE* (five items: e.g., “I feel used up at the end of the workday”), *CY* (five items: e.g., “I have become less enthusiastic about my work”), and *rPE* (six items: e.g., “In my opinion, I am good at job,” reverse coded). High scores in EE refer to feelings of being emotionally exhausted by work (chronic fatigue). High scores in CY mean that individuals report on a negative, hostile, or extremely detached attitude toward work. High scores in rPE are related to feelings of incompetency and unsuccessful achievement at the work place. Items were answered on a seven-point fully anchored Likert scale ranging from 0 (never) to 6 (every day). Consequently, each burnout score varied between 0 and 6, with values close to the high anchor indicating that these burnout symptoms are experienced every day. Besides an item mean score of each burnout dimension, an overall burnout score was conducted using the algorithm proposed by Kalimo et al. (2003) (0.4^∗^EE+0.3^∗^CY+0.3^∗^rPE; see Kalimo et al., 2003).

Need for cognition was measured using the 16-item version of the German NFC-scale (Bless et al., 1994). Responses on items such as “Thinking is not my idea of fun” (reverse coded) were given on a 7-point Likert scale ranging from -3 (completely inappropriate) to 3 (absolutely appropriate). Thus, the NFC sum score can range from -48 to + 48, with more positive scores indicating a greater tendency to engage in and enjoy cognitive activity.

The FFM was measured by using the 10-item short version of the Big Five Inventory (BFI-10, Rammstedt and John, 2007). It measures the five personality factors N, E, O, A, and C with two items for every factor. Answers to items such as “I see myself as someone who gets nervous easily.” (N) were recorded on a five-point Likert scale ranging from 1 (disagree strongly) to 5 (agree strongly) and the mean of the two answers was calculated for each dimension.

#### Statistical Analyses

The statistical analyses were performed using IBM SPSS Statistics 23 (SPSS Inc., Chicago, IL, United States). In a first step, Pearson correlation coefficients were applied to examine the relationship of NFC and the FFM with the three dimensions of the MBI as well as the total MBI score. To disentangle variance of the considered personality factors and to examine the contribution of NFC in explaining variance in the burnout symptoms over and above the FFM, in addition, hierarchical regression analyses were computed, with each of the MBI sub-dimensions and the MBI total score as criterion and the FFM (first step) and additionally the NFC sum score (second step) as predictors. Due to multiple testing, a Bonferroni corrected significance level was used.

### Results

#### Descriptives

##### Burnout

Table 1 contains the mean values, standard deviations, internal consistencies (Cronbach’s α), and intercorrelations of all variables. Internal consistencies of the MBI sub-dimensions ranged between 0.82 and 0.90 and were thus good to excellent. The MBI sub-dimensions were moderately to highly related. Highest correlation was observed between MBI-EE and CY (*r* = 0.61), followed by MBI-CY and rPE (*r* = 0.44) and EE and rPE (*r* = 0.31).

**Table 1:** Descriptives and intercorrelations of the MBI- and personality factors.

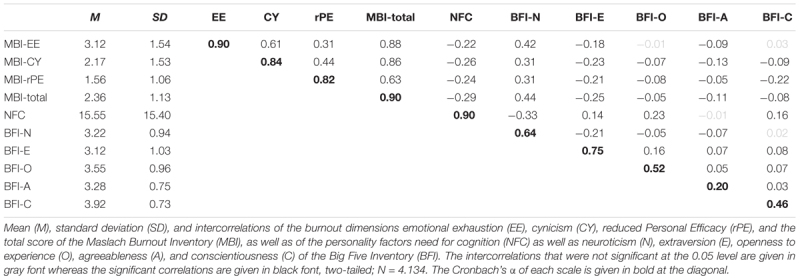

##### Personality measures

Internal consistency of the NFC scale was excellent (Cronbach’s α = 0.90) while for the BFI dimensions lower values were observed (α = 0.64, 0.75, 0.52, 0.20, and 0.46 for N, E, O, A, and C) as usually found for short scales assessing broad constructs (Ziegler et al., 2014). In accordance with previous studies (e.g., Fleischhauer et al., 2010), NFC was negatively related with N (*r* = -0.33) and positively with E (*r* = 0.14), O (*r* = 0.23), and C (*r* = 0.16) (Table 1).

##### Personality and burnout

The main purpose of the present study was to examine the role of NFC in explaining variance in burnout symptoms over and above the FFM. As depicted in Table 1, NFC showed significant small to moderate correlations with each of the MBI subscales (*r* = -0.22 for MBI-EE, *r* = -0.26 for MBI-CY, and *r* = -0.24 for MBI_rPE) and a moderate association with the MBI total score (*r* = -0.29) (Cohen, 1988).

With respect to the Big-Five dimensions, as previously reported (Alarcon et al., 2009; Swider and Zimmerman, 2010), N showed moderate associations with the EE scale of the MBI (*r* = 0.42) and to a smaller, but still moderate degree with CY (*r* = 0.31) and rPE (*r* = 0.31). For *BFI-E*, significant negative associations with the MBI factors (*r* = -0.18 to -0.25) were observed. With respect to *BFI-O*, only small correlations occurred for the total MBI-score and the sub-dimensions CY and rPE (*r* = -0.05 to -0.08). Similarly, for *BFI-A*, small correlations (-0.05 to -0.13) were found for all MBI factors. Finally, for *BFI-C*, there was a small negative association with the MBI total factor (*r* = -0.08), which was mainly driven by the MBI sub-dimension rPE (*r* = -0.22) and CY (*r* = -0.09). Using a conservative Bonferroni-corrected *p*-value that takes the number of single comparisons into account (0.05/24 = 0.002), all these correlations remained significant.

##### Association between NFC and the MBI factors controlled for the FFM measures

In a next step, we conducted hierarchical regression analyses to investigate the incremental value of NFC in explaining variance in the MBI measures (EE, CY, rPE, and the total MBI score) over and above the FFM. To account for multiple testing, the significance level was again Bonferroni-corrected by dividing the conventional significance level by the number of models considered (0.05/4 = 0.0125). Because age and sex were significantly associated with the dependent and independent variables (maximum *r* = 0.18 between age/sex and BFI-C), we included age and sex additionally to the FFM measures in a first step and then added NFC in a second step. When changes in *R*^2^ from step 1 (age, sex, and FFM measures as predictors) to step 2 (age, sex, FFM, and NFC as predictors) met the corrected significance level, NFC was interpreted as incremental predictor of burnout.

As depicted in Table 2, NFC significantly explained variance in all four MBI measures over and above the FFM. For the total MBI score and CY, NFC explained 2% more in variance (*p* < 0.001). The gain for EE and rPE was 1% (*p* < 0.001). Note that the variance in CY, rPE, and the total MBI-score that was additionally explained by NFC was 1% higher when only those individuals were considered who reported to be mainly cognitively engaged at work (*n* = 3.113). In contrast, for individuals who reported to be mainly physically engaged or both, physically and cognitively in equal terms, NFC showed incremental validity only regarding the total MBI score (Δ*F* = 6.59, *p* = 0.01) whereas change in *R*^2^ was not significant with respect to the models including the burnout facets (all *p* > 0.0125).

**Table 2:** Prediction of MBI dimensions by FFM and NFC.

Criterion	MBI-total		MBI-EE		MBI-CY		MBI-rPE	
Step	*β*	*p*	*β*	*p*	*β*	*p*	*β*	*p*
1. age	0.01	0.164	0.07	<0.001	–0.01	0.453	–0.05	0.001
sex	–0.06	<0.001	–0.02	0.145	–0.07	<0.001	–0.05	0.001
BFI-N	0.41	<0.001	0.40	<0.001	0.28	<0.001	0.30	<0.001
BFI-E	–0.15	<0.001	–0.10	<0.001	–0.15	<0.001	–0.13	<0.001
BFI-O	0.01	0.738	0.03	0.042	–0.01	0.242	–0.02	0.126
BFI-A	–0.07	<0.001	–0.06	<0.001	–0.09	<0.001	–0.01	0.435
BFI-C	–0.07	<0.001	0.02	0.186	–0.06	<0.001	–0.20	<0.001
*R*^2^	0.24	<0.001	0.19	<0.001	0.15	<0.001	0.17	<0.001
2. age	0.02	0.173	0.07	<0.001	–0.01	0.420	–0.05	0.001
sex	–0.06	<0.001	–0.03	0.062	–0.08	<0.001	–0.06	<0.001
BFI-N	0.37	<0.001	0.36	<0.001	0.23	<0.001	0.26	<0.001
BFI-E	–0.14	<0.001	–0.09	<0.001	–0.15	<0.001	–0.12	<0.001
BFI-O	0.04	0.009	0.05	0.001	0.02	0.144	0.00	0.997
BFI-A	–0.08	<0.001	–0.06	<0.001	–0.10	<0.001	–0.02	0.250
BFI-C	–0.05	0.001	0.04	0.012	–0.03	0.022	–0.18	<0.001
NFC	–0.16	<0.001	–0.11	<0.001	–0.16	<0.001	–0.11	<0.001
*R*^2^	0.26	<0.001	0.20	<0.001	0.17	<0.001	0.18	<0.001
Change in *F*	**108.19**	**<0.001**	**50.87**	**<0.001**	**106.03**	**<0.001**	**45.80**	**<0.001**


*The burnout dimensions of the MBI emotional exhaustion (EE), cynicism (*CY*), reduced Personal Efficacy (*rPE*), and the total score were regressed on age, sex, and the personality factors neuroticism (N), extraversion (E), openness to experience (O), agreeableness (A), and conscientiousness (C) of the Big Five Inventory (BFI) in Step 1 and additionally on need for cognition (NFC) in Step 2 (*N* = 4.134). All changes in *R*^*2*^ from Step 1 to Step 2 that met the Bonferroni-corrected significance level of *p* = 0.05/4 = 0.0125 are depicted in bold.*

### Discussion

Based on a population wide sample of 4.134 individuals, we examined the association of NFC and FFM with the MBI facets EE, CY, and rPE. NFC showed small to moderate negative associations with all burnout facets. With respect to the FFM as measured with the BFI-10 (Rammstedt and John, 2007), moderate positive associations were observed between N and the three burnout facets while the other BFI dimensions showed no to small negative associations with these facets. Since NFC and the FFM factors overlap to some parts, we further examined whether the associations between NFC and the MBI facets remain stable when the shared variance with the FFM was controlled for. In the hierarchical regression analyses, NFC explained variance in all burnout facets over and above the FFM. This incremental validity of NFC demonstrates that the cognitive motivation of individuals may play a crucial role in coping with work-related demands. That is, the positive attitudes and feelings toward cognitively demanding tasks of individuals high in NFC may partly protect them from work-related stress and thus from experiencing burnout symptoms.

However, the incremental value of NFC in explaining variance in the burnout facets over and above the FFM was rather small in nature (around 2%). This might be related to the population-wide sample examined, which was heterogeneous in socio-demographic factors as well as in stress-factors. That is, work-related stress factors are due not only to cognitive demands but also to emotional, physical, and other demands. NFC, however, should be especially relevant when facing cognitive demands. First evidence for this assumption was derived from additional analyses showing that the incremental validity of NFC in explaining burnout variance could be improved when only individuals were considered that reported to be mainly cognitively engaged at work. That is, investigating the relationship between NFC and burnout factors in a sample that is more homogenous in experiencing cognitive stress factors may lead to more pronounced associations.

Moreover, because data collection had to follow the prerequisite of economic assessment to minimize self-selection and thus to realize a most representative sample, a very short form of the BFI was used for which low internal consistency was observed at least regarding the O, A, and C scales. As discussed by Rammstedt et al. (2013), internal consistency is not a good estimate of reliability for the BFI-10 as the two items of each dimension are heterogeneous to depict the breadth of content of each Big-Five dimension (see also Ziegler et al., 2014). That is, because the Cronbach’s α coefficient takes into account the number of items, a high internal consistency is almost impossible for the BFI-10 or goes along with a redundancy of the two items. Instead, Rammstedt and colleagues used retest-reliability and obtained satisfactory coefficients for all dimensions of the BFI-10. Moreover, they showed that the low reliability does not go along with low validity as the BFI-10 showed structural validity as well as convergent validity with peer ratings and longer Big-Five measures (Rammstedt and John, 2007; Rammstedt et al., 2013). Nevertheless, in Study 2, a more reliable measure was used to assess the FFM and a more homogenous student sample was considered in which stress-factors are especially cognitive in nature.

## Study 2

In Study 2, we investigated the relationship between NFC and burnout symptoms in a more homogenous student sample. Although such a sample may suffer from the problem of restriction of range, this sample has the advantage of being more homogeneous regarding age, occupation, and life circumstances, variables that may confound the relationship between cognitive motivation and burnout.

Furthermore, we examined whether NFC may play a moderating role for the inconsistently found negative association between burnout and executive functioning in non-clinical samples. Because NFC refers to high intrinsic cognitive motivation to invest cognitive resources/effort and to engage in and enjoy cognitively challenging endeavors (for an overview, see Cacioppo et al., 1996; see also Enge et al., 2008; Strobel et al., 2015), one may expect that NFC attenuates the negative influence of burnout on cognitive functioning.

### Materials and Methods

#### Participants and Procedure

The sample comprised 127 students of psychology (39%) and other subjects at the Technische Universität Dresden (74% female; *mean* age = 23.61, *SD* = 4.1 years, range: 18–40 years). Participants gave written informed consent and received either monetary rewards (18€) or course credit for their participation. Participants worked on several questionnaires and three executive function tasks measuring inhibition, shifting, and working memory updating (for task description, see below) once in the laboratory and once at home with 1 week in between and in a randomized order because a further goal was to measure whether task performance differed between home compared to the lab which is reported elsewhere (Miller et al., 2018). There were no substantial differences between the lab and home context. For reasons of comparability with Study 1 and because there was no significant change in burnout scores (all *p* > 0.05), we considered only the data collected during the first measurement.

All tasks had been completed on a computer using the software Inquisit (Millisecond Software LLC, 2006). After socio-demographics (age, sex, and study subject) were assessed, participants completed the following personality questionnaires and executive tasks in the given order: 1. NFC scale, 2. Number–letter task, 3. MBI, 4. Go/NoGo task, 5. BFI-K, and 6. two-back task.

#### Measures

*Burnout* scores were measured with the German MBI student version (MBI-SS; Schaufeli et al., 2002). The 15 items of the three dimensions EE (five items, e.g., “I feel used up at the end of a day at university”), CY (four items, e.g., “I have become less enthusiastic about my study”), and rPE (six items, e.g., “In my opinion, I am a good student” – invers coded) were answered on a fully anchored 7-point Likert scale ranging from 0 (never) to 6 (daily).

As in Study 1, *NFC* was assessed by the 16-item German NFC scale (Bless et al., 1994). To measure the *FFM*, participants filled in the Big-Five Inventory (BFI-K, short form of the BFI) by Rammstedt and John (2005). The five factors E, N, O to Experiences, A, and C are measured with 21 items (four items for N, E, A, and C; five for O). The items such as “I get nervous easily.” (N) have to be answered on a five-point-Likert scale ranging from 1 (very inappropriate) to 5 (very appropriate).

Moreover, three tasks assessing executive functions were conducted. During the *number–letter task* measuring the shifting function, participants had to respond to either numbers or letters depending on whether they were presented above or below a horizontal line. In every trial, a pair of the letters “a” or “b” and the numbers “1” or “2” appeared (e.g., “a1”). If the number–letter pair was presented above the line, the participants had to respond to the numbers. If it appeared below the line, they had to respond to the letters. For “a” and “1,” participants had to press the key “Y.” For “b” and “2,” they had to press the key “M” at the computer pad.

In the *Go*/*NoGo Task* measuring response inhibition, participants were required to classify the alignment of two circles as vertical (predominant go-stimulus) or horizontal (no-go stimulus). For go-stimuli that were presented seven times more often than no-go stimuli, the key “M” had to be pressed. No-go stimuli had to be classified by the key “Y.”

In the *two-Back-Task* measuring the working memory function, participants had to indicate whether a stimulus was the same as two trials before. Nine small circles of which one was marked with a cross were arranged in the form of one bigger circle. Participants had to memorize the position of the cross-marked circle. They had to press the key “Y” if the cross-marked circle appeared at the same position as two trials before (target). If not, they had to press the key “M” (non-target). Each position was presented for 2.5 s. Each of the cognitive tasks started with a short training period and lasted about 10 min (for a more detailed task description, see Miller et al., 2018).

#### Statistical Analyses

Using IBM SPSS Statistics 23 (SPSS Inc., Chicago, IL, United States), the correlations between the personality factors and the MBI facets were calculated and it was examined by hierarchical regressions whether potential associations between NFC and MBI remained stable, when the shared variance of NFC with the FFM was controlled for.

Moreover, using RStudio (RStudio Team, 2015), the role of NFC as moderator of the relationship between burnout symptoms and cognitive control functions was investigated. With respect to accuracy rate and RT (on correct trials) as dependent variables, three regression models were performed considering switch trials (Number–letter task), targets (two-back task), and nogo trials (Go/NoGo task), respectively, as criterion that was regressed on NFC, the MBI total score, and the interaction term of NFC and MBI. The two predictors were centered before the interaction term was calculated.

### Results

#### Descriptives

##### Burnout symptoms

Table 3 contains descriptives, reliabilities, and intercorrelations of all variables used in Study 2. As in Study 1, the MBI sub-dimensions and the Total score showed good to excellent internal consistencies with Cronbach’s α ranging from 0.82 (rPE) to 0.92 (CY). The MBI sub-dimensions were moderately related with highest correlation between CY and rPE (*r* = 0.48), followed by EE and CY (*r* = 0.33) and EE and rPE (*r* = 0.23).

**Table 3:** Descriptives and intercorrelations of the MBI-, personality, and executive-control measures.

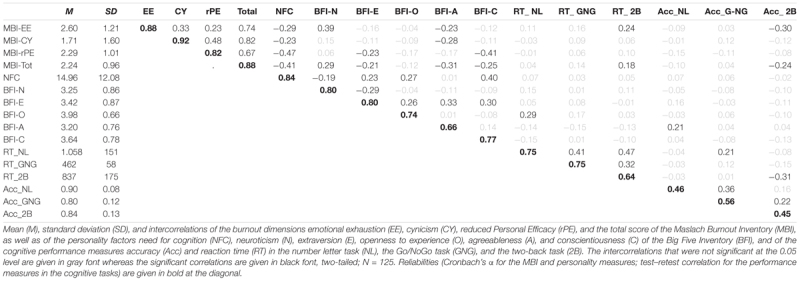

##### Personality measures

Cronbach’s α of the personality measures were good regarding NFC (α = 0.84), N (α = 0.80), and E (α = 0.80) and acceptable for O (α = 0.74), A (α = 0.66), and C (α = 0.77). Moreover, as previously shown (see e.g., Fleischhauer et al., 2010) and consistent with Study 1, a negative correlation was observed between NFC and N (*r* = -0.19), whereas NFC was positively related to C (*r* = 0.40), O (*r* = 0.27), and E (*r* = 0.23) (all *p* < 0.05).

##### Personality and bxurnout

Next, we investigated the relationship between the personality factors and burnout symptoms. As depicted in Table 3, NFC was negatively related to all burnout facets (rPE: *r* = -0.47, EE: *r* = -0.29, and CY: *r* = -0.23) and the MBI total score (*r* = -0.41). Except CY, all correlations remained significant when a conservative Bonferroni-corrected significance level (*p* = 0.002) was used to account for multiple testing (*p* = 0.05/24 comparisons).

Regarding the FFM and using the corrected significance level (*p* = 0.002), as in Study 1, N was strongly related to EE (*r* = 0.39) and to the MBI total score (*r* = 0.29) while no significant associations occurred with CY and rPE, respectively (both *p* > 0.002). Also similar to Study 1, C showed a substantial negative correlation with rPE (*r* = -0.41). Moreover, A was significantly negative related to CY (*r* = -0.28) and the MBI total score (*r* = -0.31). For E and O, no significant correlations with the MBI scales were observed (all *p* > 0.002).

##### Association of NFC and MBI controlled for the FFM measures

As in Study 1, we conducted hierarchical regression analyses to investigate the incremental value of NFC concerning the MBI dimensions over and above the FFM. Beside the FFM (Step 1) and NFC (Step 2), age and sex were included as predictors. Again, the significance level was Bonferroni-corrected by considering the number of models conducted (0.05/4 = 0.0125). When changes in *R*^2^ from Step 1 (age, sex, and FFM as predictors) to Step 2 (age, sex, FFM, and NFC as predictors) met the corrected significance level, NFC was interpreted as incremental predictor of burnout.

As depicted in Table 4, NFC explained substantial variance over and above the FFM in the total MBI burnout score (change in *R*^2^ = 0.08, *p* < 0.0125), as well as in EE (change in *R*^2^ = 0.05, *p* < 0.0125) and in rPE (change in *R*^2^ = 0.08, *p* < 0.001). Thus, similar to Study 1, NFC showed incremental validity for explaining burnout symptoms while the amount of additional explained variance was much higher than in Study 1. However, to be able to compare the results of both studies, it is necessary to show that the items of the MBI in the general version and the student version that differ in the situational context they refer to are indicators of the same latent constructs. Therefore, we additionally tested for measurement invariance of the MBI between the two samples. As outlined in the Supplementary Material, factor structure of the MBI is comparable in both samples and thus differences and similarities in the results regarding the associations between the MBI and personality measures are not a mere method artifact.

**Table 4:** Prediction of burnout dimensions by FFM and NFC.

	MBI_Total		MBI_EE		MBI_CY		MBI_rPE	
Step	β	*p*	β	*p*	β	*p*	β	*p*
1. age	0.00	0.982	0.00	0.984	0.05	0.599	–0.07	0.428
Sex	–0.04	0.726	0.06	0.575	–0.06	0.590	–0.11	0.257
BFI-N	0.27	0.003	0.35	<0.001	0.16	0.097	0.05	0.583
BFI-E	0.06	0.531	0.04	0.689	0.09	0.409	–0.01	0.950
BFI-O	–0.14	0.109	–0.05	0.601	–0.11	0.244	–0.20	0.022
BFI-A	–0.24	0.012	–0.21	0.033	–0.23	0.024	–0.07	0.472
BFI-C	–0.21	0.018	–0.09	0.322	–0.08	0.384	–0.40	<0.001
*R*^2^	0.22	<0.001	0.19	0.001	0.11	0.046	0.24	<0.001
2. age	0.00	0.982	–0.01	0.956	0.00	0.612	–0.07	0.379
Sex	–0.05	0.599	0.05	0.646	–0.04	0.528	–0.13	0.178
BFI-N	0.21	0.018	0.30	0.001	0.10	0.203	–0.01	0.894
BFI-E	0.06	0.525	0.04	0.693	0.08	0.411	–0.01	0.929
BFI-O	–0.04	0.659	0.03	0.730	–0.05	0.626	–0.10	0.255
BFI-A	–0.27	0.004	–0.23	0.017	–0.28	0.015	–0.09	0.295
BFI-C	–0.07	0.493	0.02	0.812	–0.10	0.952	–0.25	0.007
NFC	–0.34	<0.001	–0.26	0.009	–0.20	0.051	–0.33	<0.001
*R*^2^	0.30	<0.001	0.24	<0.001	0.14	0.020	0.32	<0.001
Change in *F*	**13.13**	**<0.001**	**7.09**	**0.009**	**3.90**	**0.051**	**13.12**	**<0.001**


*To investigated the incremental value of NFC in explaining variance in burnout symptoms, the burnout dimensions of the MBI emotional exhaustion (EE), cynicism (*CY*), reduced Personal Efficacy (*rPE*), and the total score were regressed on age, sex, and the personality factors neuroticism (N), extraversion (E), openness to experience (O), agreeableness (A), and conscientiousness (C) of the Big Five Inventory (BFI) in Step 1 and additionally on NFC in Step 2 (*N* = 125). Values depicted in bold refer to all changes in *F*-statistics that met the Bonferroni-corrected significance level of *p* < 0.0125 (0.05/4 models; *N* = 125).*

##### Effects of NFC on the link between burnout score and cognitive functioning

Next, we investigated whether NFC plays a moderating role for the relationship between the MBI burnout score and the cognitive performance measures. As depicted in Table 3, there were only small to moderate associations of the MBI total score and the sub-dimension EE with RT and accuracy in the two-back task (*r* = |0.18| to |0.30| ) while for the Number–letter and the Go/NoGo tasks, no correlation with the MBI measures occurred.

As, however, effects of burnout symptoms on cognitive functioning may only occur when relevant moderator variables such as NFC are considered, moderated regression analyses were performed with the two performance measures (accuracy rate and RT) in the three cognitive control tasks as criterion and with the MBI total score and NFC as well as the interaction term of MBI and NFC as predictors. As we ran six regression analyses (three tasks, two criteria) with three predictors each (two main effects and one two-way interaction), the Bonferroni-adjusted level of significance was set to *p* = 0.05/18 ∼ 0.003.

With respect to the two-back task, the MBI total score significantly predicted accuracy, *B* = -0.04, *p* = 0.001, as well as RT, *B* = 47.3, *p* = 0.001, indicating lower accuracy and higher RT rates observed for individuals with higher MBI scores. In none of the models, NFC or the interaction of NFC × MBI was a significant predictor (all *p* > 0.003).

### Discussion

In Study 2, we investigated the relationship of NFC and FFM with the MBI facets EE, CY, and rPE in a more homogenous sample of 125 students. As study-related stress factors of students are more cognitive in nature, we expected especially NFC to be negatively related to burnout symptoms as a high cognitive motivation and a higher preference and enjoyment for cognitively demanding than cognitively simple tasks (Cacioppo and Petty, 1982; See et al., 2009; Fleischhauer et al., 2015) might be good prerequisites to tolerate cognitive stress factors.

As expected and similar to Study 1, NFC showed negative associations with the MBI total score, which was especially due to the MBI sub-dimension rPE. Similar associations were only observed between rPE and C. When commonly examined in a multiple linear regression, however, both factors contributed equally to the variance in rPE indicating that their influence is not due to shared variance between NFC and C. Moreover, there was a negative association between NFC and the MBI facet EE that gives a significant contribution over and above the frequently reported association between N and MBI-EE (Alarcon et al., 2009; Swider and Zimmerman, 2010). Given the effect sizes, NFC appeared to be the most relevant personality factor in explaining variance in MBI symptoms in this young and non-clinical student sample.

Moreover, we examined whether burnout is associated with cognitive impairments as found in several studies (see Deligkaris et al., 2014; Table 2), and whether NFC might be a moderating factor of this relationship. In our student sample, a negative association between burnout and cognitive performance was observed for the two-back task measuring working memory updating. Here, individuals with higher MBI scores showed lower accuracy rates and higher RTs than those who reported lower burnout scores. This seems to fit with a recent systematic meta-analysis evaluating the role of burnout in inhibition, switching/shifting, and updating function in non-clinical and clinical samples (Deligkaris et al., 2014). Here, the five studies assessing the updating function consistently found burnout to be associated with cognitive impairments, whereas for the other two executive functions the results were mixed. For shifting four of five studies and for inhibition four of six studies showed the expected negative associations, while the other studies revealed no effects. Similarly, in our study, burnout was not associated with inhibition or shifting performance. While in the meta-analysis mainly clinical samples were considered, our sample consists of young non-clinical students who are characterized by high performance and integrity of cognitive control functions. Thus, we not only examined a sample with less severe burnout symptoms [the mean total MBI score was 2.24 while Kalimo et al. (2003) determined the cut-off of severe burnout symptoms at values ≥3.50,see Kalimo et al., 2003], but also with a higher ability to compensate for cognitive stress which might explain the lower effect sizes of our study compared to studies summarized by Deligkaris et al. (2014). This, in turn, might also be the reason for the lacking interaction effect of NFC and burnout symptoms on cognitive performance measures.

## General Discussion

The present research dealt with the question whether NFC as a marker of individual differences in the tendency to engage in and enjoy cognitive activity (Cacioppo and Petty, 1982) may contribute to resilience against burnout symptoms. This assumption was derived from the fact that individuals high in NFC enjoy effortful cognitive endeavors, search for cognitive engagement, and prefer complex to simple tasks (Cacioppo and Petty, 1982; See et al., 2009; Fleischhauer et al., 2015). Accordingly, they should better cope with stress especially in a cognitively demanding context, including the working place context, which is increasingly characterized by cognitive challenges. Indeed, in two independent samples we observed NFC to be negatively associated with burnout symptoms. In the large population-wide sample of Study 1 (*N* = 4.134), NFC showed small to moderate negative associations with the three burnout symptoms EE, CY, and rPE which remained significant when shared variance with the FFM that shows substantial correlations with burnout symptoms (Alarcon et al., 2009; Swider and Zimmerman, 2010) and with NFC (see e.g., Fleischhauer et al., 2010) was controlled for. One might argue that due to their higher intrinsic motivation to deal with complex cognitive tasks (Cacioppo et al., 1996; See et al., 2009), individuals high in NFC should rather not feel stressed when confronted with a cognitively demanding task at work, but rather enjoy tasks that are cognitively challenging. In contrast, those low in NFC should rather have difficulties to cope with cognitively challenging situations due to their lower enjoyment and motivation for cognitive endeavors. A higher cognitive load especially for longer periods may be accompanied by negative emotions which in turn may be reflected in the negative correlations between NFC and EE and CY. Moreover, NFC is conceptualized as being rather process-oriented than -oriented. That is, those high in NFC enjoy cognitively demanding tasks relatively independent of whether they are indeed successful or not (Cacioppo et al., 1996). As follows, they may experience less frustration in the latter situations, which might additionally explain the negative association between NFC and rPE.

In the more homogenous sample of young students in Study 2, a similar pattern of results was observed. Although due to the smaller sample size, the correlation with CY did not reach significance. However, in the student sample, NFC showed much higher association with rPE. Moreover, when examined together with the FFM in a multiple regression model, NFC explained more additional variance in EE and rPE in the student sample (gain of 5 and 10%) than in the broad population-wide sample (gain of 2% at maximum). As already discussed above, study-related stress in the student sample is typically more related to cognitive challenges. In contrast, jobs can be more or less cognitively demanding (e.g., a job as product manager should be more cognitively demanding than a job in a call center that may on average be more emotionally demanding). Moreover, work-related stress may be associated with more heterogeneous sources of stress (see Maslach et al., 2001; Kaschka et al., 2011), resulting not only from the content of work (e.g., emotion work with clients), but also from working conditions (e.g., physical working conditions, amount of control and autonomy, role ambiguity).

However, the found differences in the strength of associations between NFC and burnout symptoms could have also been due to the different burnout measures used in Study 1 and Study 2. The items of both measures are very similar, but while the MBI-GS (Büssing and Glaser, 1999) refers to the general working context, the MBI in the student version (Schaufeli et al., 2002) used in Study 2 stronger refers to study-related situations. To test for group invariance, we determined whether the measurement models were equivalent across the two samples (see Supplementary Material). The analyses revealed that factor structure of the MBI is comparable in both samples. That is, differences and similarities in the results are not a mere method artifact, but might relate to differences in the situational context that allows personality to exert its influence to a more or less extent. With respect to the study context, the pressure to achieve good grades at university might be the most relevant stress factor and especially traits that are responsive to cognitively demanding situations should be related to burnout symptoms. While individuals who enjoy investing cognitive resources (high NFC) may better cope with these demands, individuals with a low NFC might experience themselves less efficient and more emotional exhausted. Support for this interpretation was also found in Study 1, where NFC explained some more variance in burnout symptoms when only individuals were considered that reported to be mostly faced with cognitive tasks at work also indicating the construct validity of NFC.

In Study 2, we additionally aimed to examine whether burnout symptoms are associated with impairments in cognitive functions which was often reported by previous research (for a meta-analtyic review, see Deligkaris et al., 2014) but appears to depend on moderating factors such as task difficulty (Diestel et al., 2013) or type of stimuli (Bianchi et al., 2018) in non-clinical samples. In our study, it was of interest, whether NFC as trait factor of cognitive motivation may moderate the relationship between burnout symptoms and executive control insofar that a negative association is observed for individuals low in NFC, but not for those high in NFC as they may be more able to compensate for burnout-related impairments. This hypothesis was also derived from research showing that individuals with higher levels of NFC report less cognitive impairments associated with depression (Nishiguchi et al., 2016) that shows large syndrome overlap with burnout (Bianchi et al., 2015). In our study, however, the MBI total score was only significantly associated with performance in the two-back task indicating working memory updating and there was no moderating effect of NFC on the relationship between burnout symptoms and performance in the executive control tasks. Given the findings of Nishiguchi et al. (2016), one might argue that NFC rather moderates the self-rated cognitive performance but not the actual performance. Moreover, the results may indicate that burnout-related impairments might be limited in our young and non-clinical sample of students also limiting a moderating role of NFC.

## Limitations and Future Research

As discussed above, one limitation of Study 2 is the use of a non-clinical sample which might have made it harder to detect burnout-related cognitive impairments and a moderating role of NFC in the relationship of burnout and cognitive impairments. Thus, to investigate this relationship in a clinical sample considering individuals that experience severe work- or study-related stress might be worthwhile. In this context, Eskildsen et al. (2016) observed that patients with work-related stress showed not only cognitive impairments compared to healthy controls at baseline, but also 1 year later. Proceeding from these findings, an interesting approach for future research would be to examine whether NFC and other personality factors might influence recovering from stress-related sick leave and cognitive impairments.

A further limitation is the use of the BFI-10 in Study 1. Its brevity (two items per subscale) resulted in low internal consistency, especially for O, A, and C. As low internal consistencies are often accompanied by low validity, associations of the FFM with burnout symptoms might have been underestimated and the incremental validity of NFC over and above the FFM might have been overestimated in Study 1. Moreover, the use of the less reliable BFI-10 in Study 1 might limit the comparability of results with those gained in Study 2 where the more reliable BFI-K was used. The overall result pattern, however, gives evidence that the negative consequences of the low internal consistency for the validity of the BFI-10 are limited. So, the direction of correlations with the MBI measures as well as the mean size was quite similar between the two BFI measures (BFI-10: mean |*r|* = 0.17; BFI-K: mean |*r|* = 0.19). This was also true regarding the intercorrelations of the BFI-dimensions with NFC (BFI-10: mean |*r|* = 0.17; BFI-K: mean |*r|* = 0.22). Here, it would have been worthwhile to have measured the BFI-10 twice in order to prove whether the retest-reliability is indeed a more appropriate indicator for reliability of very short-scales in our sample (see Rammstedt et al., 2013; Ziegler et al., 2014).

Finally, the cross-sectional approach of both studies limits the interpretation of the results. Thus, we cannot rule out the possibility of a reversed or bi-directional causality effect. Consequently, longitudinal approaches are needed to investigate the causal relationship of NFC on burnout symptoms. With respect to the FFM, there are a few studies that investigated longitudinal associations between burnout and the FFM. In a sample of nursing students, Deary et al. (2003) showed that N at T1 was significantly positively related to the burnout facet EE assessed at T2 (12 month after T1), but not to EE at T3 (24 month after T1). Similarly, in a large representative sample of *N* = 1.105 participants, Armon et al. (2012) showed that N positively predicted global burnout at T1, but that the association was not stable over a time lag of 24 months. Concerning the burnout facet EE, however, N negatively predicted EE at T1 and T2. Woods et al. (2013) argued that individuals high in N might “withdraw from threatening situations that might lead to EE” which could explain the found result pattern by Deary et al. (2003) and Armon et al. (2012). Accordingly, individual differences in personality might influence how occupational experiences are evaluated and how individuals respond to these experiences. Likewise, situational factors such as occupational environment might shape personality. For example, an individual experiencing continuing time pressure at work or at university may experience her- or himself as not being able to elaborate on information, and so may develop a lower NFC self-concept. To investigate such dynamics and to reveal situational-cognitive factors that contribute to the found correlations between personality and burnout remains a challenge for future research.

## Data Availability

The datasets generated for this study are available on request to the corresponding author.

## Ethics Statement

All subjects gave written informed consent in accordance with the Declaration of Helsinki. The study protocol was approved by the Ethics Committee of the Technische Universität Dresden Medical School.

## Author Contributions

CK, RM, SE, and MF conceived and designed the studies with respect to the specific research questions. MF, SE, RM, MW, and MP conducted the studies. MF and SE analyzed and interpreted the data and supervised the work. MF mainly wrote and SE, MW, and MP wrote parts of the paper and commented on the manuscript.

## Conflict of Interest Statement

The authors declare that the research was conducted in the absence of any commercial or financial relationships that could be construed as a potential conflict of interest.
