# Supplementary material for: Thinking Against Burnout? An Individual’s Tendency to Engage in and Enjoy Thinking as a Potential Resilience Factor of Burnout Symptoms and Burnout-Related Impairment in Executive Functioning
Source: Front Psychol. 2019 Mar 12;10:420. doi: 10.3389/fpsyg.2019.00420 (PMC6422970; doi:10.3389/fpsyg.2019.00420)
Supplement: Supplementary file 1 [file Table_1.DOCX]

**Additional analysis of measurement invariance of the MBI scores in Study 1 and 2**

The R-package semTools ([Pornprasertmanit, Miller, Schoemann, & Rosseel, 2013](#_ENREF_5)) was used to test for measurement invariance between the general version of the MBI ([MBI-GS, Büssing & Glaser, 1999](#_ENREF_1)) used in Study1 and the student version of the MBI ([MBI-SS, Schaufeli, Martinez, Pinto, Salanova, & Bakker, 2002](#_ENREF_6)) used in Study 2. The MBI-GS was shortened by one item of the CY-subscale that was not existent in the MBI-SS. Note, that we also checked whether the associations between personality and CY reported in Study 1 changed when the shortened subscale was used. All observed associations as well as the incremental value of NFC in explaining variance in CY remained significant and effect sizes were nearly the same.

First, a confirmatory factor analysis was performed with the three factor-solution proposed by Maslach and Jackson ([1981](#_ENREF_4)). The factors were allowed to correlate with each other. The fit indices for this model were: *𝟀²* (87) = 2609.95, *p* < 0.001, *CFI* = 0.922, *RMSEA* = 0.083, *SRMR* = 0.055, indicating acceptable model fit.

A step-wise procedure of model testing was used. In a first step a baseline model that allows the free estimation of the item loadings (configural invariance) was compared with a more restrictive model forcing all item loadings to be equal between the two samples (metric invariance). Then, the metric model was compared with a more restrictive model that additionally forces the intercepts to be equal in both samples (scalar invariance). Finally, it was tested whether model fit changed when additionally the residuals were forced to be invariant (residual invariance). As recommended by Cheung and Rensvold ([2002](#_ENREF_3)), a decrease in *CFI* of ≤ 0.01 and an increase in *RMSEA* of ≤ 0.015 were evaluated as indicating invariance ([Chen, 2007](#_ENREF_2)). The measurement invariance test showed invariance on factor loading level (*ΔCFI*=0.001, *ΔRMSEA*=0.002), on intercept level (*ΔCFI*=0.009, *ΔRMSEA*=0.001) and also on the residual level (*ΔCFI*=0.001, *ΔRMSEA*=0.002). Thus, the factor structure of the MBI is comparable in both samples and thus differences and similarities in the results regarding the associations between the MBI and personality measures are not a mere method artifact.

**References**

Büssing, A., & Glaser, J. (1999). Deutsche Fassung des Maslach burnout inventory–General survey (MBI-GS-D). *Munich: München, Technische Universität, Lehrstuhl für Psychologie*.

Chen, F. F. (2007). Sensitivity of goodness of fit indexes to lack of measurement invariance. *Structural equation modeling, 14*(3), 464-504.

Cheung, G. W., & Rensvold, R. B. (2002). Evaluating goodness-of-fit indexes for testing measurement invariance. *Structural equation modeling, 9*(2), 233-255.

Maslach, C., & Jackson, S. E. (1981). The measurement of experienced burnout. *Journal of Organizational Behavior, 2*(2), 99-113.

Pornprasertmanit, S., Miller, P., Schoemann, A., & Rosseel, Y. (2013). semTools: Useful tools for structural equation modeling. *R package version 0.4–14*.

Schaufeli, W. B., Martinez, I. M., Pinto, A. M., Salanova, M., & Bakker, A. B. (2002). Burnout and engagement in university students: A cross-national study. *Journal of Cross-Cultural Psychology, 33*(5), 464-481.
